# Mathematical modeling of translation initiation for the estimation of its efficiency to computationally design mRNA sequences with desired expression levels in prokaryotes

**DOI:** 10.1186/1752-0509-4-71

**Published:** 2010-05-26

**Authors:** Dokyun Na, Sunjae Lee, Doheon Lee

**Affiliations:** 1 Department of Bio and Brain Engineering, KAIST, 335-Gwahangno, Yuseong-gu, Daejeon 305-701, Republic of Korea; 2 Bioinformatics Research Center, KAIST, 335-Gwahangno, Yuseong-gu, Daejeon 305-701, Republic of Korea

## Abstract

**Background:**

Within the emerging field of synthetic biology, engineering paradigms have recently been used to design biological systems with novel functionalities. One of the essential challenges hampering the construction of such systems is the need to precisely optimize protein expression levels for robust operation. However, it is difficult to design mRNA sequences for expression at targeted protein levels, since even a few nucleotide modifications around the start codon may alter translational efficiency and dramatically (up to 250-fold) change protein expression. Previous studies have used *ad hoc *approaches (e.g., random mutagenesis) to obtain the desired translational efficiencies for mRNA sequences. Hence, the development of a mathematical methodology capable of estimating translational efficiency would greatly facilitate the future design of mRNA sequences aimed at yielding desired protein expression levels.

**Results:**

We herein propose a mathematical model that focuses on translation initiation, which is the rate-limiting step in translation. The model uses mRNA-folding dynamics and ribosome-binding dynamics to estimate translational efficiencies solely from mRNA sequence information. We confirmed the feasibility of our model using previously reported expression data on the MS2 coat protein. For further confirmation, we used our model to design 22 *luxR *mRNA sequences predicted to have diverse translation efficiencies ranging from 10^-5 ^to 1. The expression levels of these sequences were measured in *Escherichia coli *and found to be highly correlated (*R*^*2 *^= 0.87) with their estimated translational efficiencies. Moreover, we used our computational method to successfully transform a low-expressing DsRed2 mRNA sequence into a high-expressing mRNA sequence by maximizing its translational efficiency through the modification of only eight nucleotides upstream of the start codon.

**Conclusions:**

We herein describe a mathematical model that uses mRNA sequence information to estimate translational efficiency. This model could be used to design best-fit mRNA sequences having a desired protein expression level, thereby facilitating protein over-production in biotechnology or the protein expression-level optimization necessary for the construction of robust networks in synthetic biology.

## Background

The emerging research field of synthetic biology differs from conventional biotechnology in terms of its problem-solving strategies [1]. Synthetic biology uses the engineering paradigm of system design to build biological systems with novel functionalities that often do not exist in nature. Therefore, synthetic biology allows the rational design or redesign of living systems at a deep and complex level [2-4], allowing researchers to use existing biological knowledge to rationally and systematically tackle biological problems.

When synthetic networks are designed, genetic regulation is considered at the level of transcription, while translation is assumed to be straightforward and is therefore ignored [5,6]. However, a few nucleotide changes around the start codon can dramatically affect translation efficiency and may alter protein expression levels by up to 250-fold [7-10]. Thus, if both transcription and translation processes are not considered during the design of synthetic networks, the realized networks could show unpredictable or unstable behavior [7,8,11,12]. In order to guarantee the robust operation of synthetic networks, the kinetics of both transcription and translation should be optimized, much in the way that nature has optimized biological systems through evolution [13,14].

The translational efficiency of an mRNA is highly dependent on the nucleotides in the translation initiation region determining the mRNA molecule's conformation and ribosome-binding affinity. Thus, it is difficult to estimate translational efficiency directly from mRNA-sequence data, and to design mRNA sequences that will be expressed at desired protein levels. Random mutagenesis of nucleotides in the translation initiation region has been widely used to tailor mRNA sequences toward desired expression levels. However, because translational efficiency is highly dependent on the downstream coding sequence, the time-consuming process of repeated mutagenesis and selection must be used to optimize the nucleotides in the translation initiation region of each coding sequence [13,15-17].

The ability to express a given protein at the desired level is key to systematically and efficiently building robust synthetic networks. Toward this end, it would be highly useful to develop a mathematical model capable of estimating the translational efficiency of mRNA sequences, thereby facilitating the rational design of useful mRNA sequences. The development of such a model would be expected to accelerate the evolution of synthetic biology.

Here we describe a model that estimates translational efficiency by focusing on the translation-initiation process which is the rate-limiting step of translation, while considering mRNA-folding dynamics and ribosome-binding affinities. To confirm the feasibility of this model, we used the MS2 coat gene as an example and compared our estimated translational efficiencies with the previously reported expression levels of the corresponding mRNA sequences. We then used our model to design *luxR *mRNA sequences in which nucleotide alterations in the translation-initiation region were predicted to yield the desired translational efficiencies, and compared these predictions with the corresponding experimental results. Finally, to show one potential application of our model, we used our model to design alterations in the translation-initiation region of the DsRed2 gene, and showed that these alterations could be used to maximize translational efficiency and transform a low-expressing DsRed2 gene into a high-expressing gene.

## Methods

**Figure 1:**
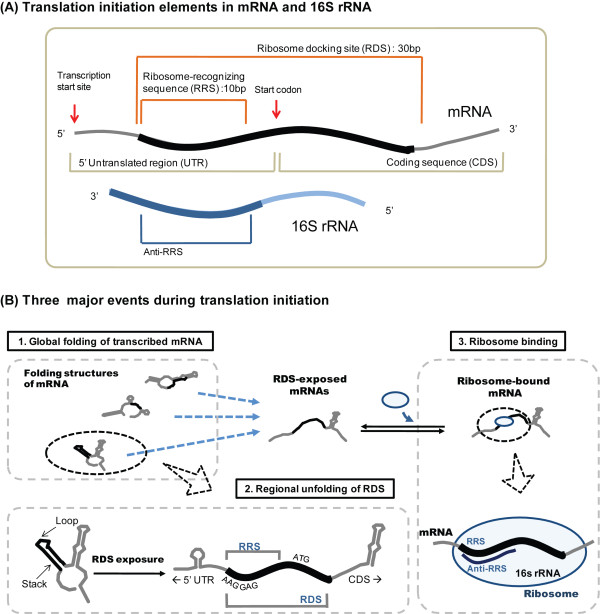
**Schematic of the translation-initiation processes included in our model**. A simple illustration of our translation-initiation model. (A) A common prokaryotic gene structure, including the parts that are essential for translation initiation. The ribosome-docking site (RDS) is the mRNA region that is actually occupied by the ribosome. Any secondary structure formed in the region can impede ribosome binding. The RDS, structurally identified by X-ray crystallography previously, starts from the first nucleotide of the Shine Dalgarno (SD) sequence, continues downstream for 30 nucleotides, and includes part of the coding sequence. The ribosome-recognizing sequence (RRS) within the RDS mediates actual binding of the ribosome to the mRNA. The strength of this binding is based on the affinity of the RRS for the anti-RRS, which is a corresponding complementary sequence found at the 3' end of the 16S rRNA (5'-*UCACCUCCUU*-3' in *Escherichia coli*). The 10-nucleotide sequence contains the conventional SD sequence (AAGGAG). (B) The translation-initiation process is modeled from mRNA folding to ribosome binding. (1) A transcribed mRNA folds into several structures according to its structural free energy. (2) Similarly, the RDS region of the mRNA is dynamically folded or unfolded according to its regional free energy. The site is exposed to ribosomes when unfolded. (3) Ribosomes bind to the exposed RDS *via *hybridization of the RRS and anti-RRS; the strength of this hybridization determines the ribosome-binding strength.

As shown in Figure 1A, we define a few new terms in this study, in order to avoid causing confusion by using conventional terms such as ribosome-binding site (RBS) or Shine Dalgarno (SD) sequence.

Translation is a sequential process of initiation, elongation, and termination. Unlike elongation and termination, initiation is a rate-limiting step that controls the overall translational efficiency [18,19]. Its efficiency is determined by various factors, including: the secondary structure of the mRNA's translation-initiation region, which is located around the start codon and mediates the translation-initiation process; and the hybridization affinity of the SD sequence in the translation-initiation region to its corresponding complementary sequence called the anti-SD sequence in the ribosome's 16S rRNA [20-27].

Briefly, during the translation-initiation process, the translation-initiation region of a transcribed mRNA dynamically folds and unfolds into and out of its secondary structure [23]. The folded structure interferes with ribosomal binding [27,28]. Once the structure is unfolded, ribosomal binding is mediated by the SD sequence based on the binding affinity between the SD and anti-SD sequences. The ribosome then incorporates the first aminoacyl-tRNA that corresponding to the start codon at the P site, thereby initiating translation. The efficiency of translation initiation is thus determined by the chance that the translation-initiation region will be unfolded, and the affinity of the SD and anti-SD sequences [23,26].

### Translation model

We herein developed a translation model that focuses on translation initiation, which is the first and rate-limiting step of translation, and pivotally facilitates the next step of translation elongation by stably attaching a ribosome to the mRNA [29]. The aim of our model is to estimate the translational efficiency of a given mRNA sequence by obtaining the probability of a given mRNA being bound to a ribosome which is directly proportional to the level of protein expression [23].

As illustrated in Figure 1B, our model includes three sequential events in initiation: (1) the thermodynamic folding of all transcribed mRNAs; (2) the regional unfolding of a given mRNA's ribosome-docking site (RDS), which is a 30-nucleotide sequence near the start codon where actual ribosome docking occurs [18,30-32]; and (3) the binding of a ribosome to the unfolded RDS through the ribosome-recognizing sequence (RRS), which is a 10-nucleotide sequence that includes the SD sequence and is complementary to a sequence at the 3' end of the 16S rRNA, termed anti-RRS (*e.g*., 5'-*UCACCUCCUU*-3' in *E. coli*) [33].

Briefly, a transcribed mRNA can fold into diverse conformations according to its structural stabilities. A partition function can be used to calculate the fraction of each conformation based on its free energy [34]. Although each conformation has a certain overall stability, any regional secondary structure (*e.g*., a helix or stem) can be dynamically folded or unfolded according to its regional stability. For a ribosome to bind, the RDS must be unfolded. In short, all nucleotides in the site must lose their base pairings. We therefore estimate the chance of regional unfolding at a ribosome-docking site, and call this the "exposure probability."

In order to recruit ribosomes, a ribosome-docking site must possess a sequence complementary to the ribosomal 16S rRNA, as this is where hybridization occurs [35]. The hybridizing sequence in the mRNA is defined as the "ribosome-recognizing sequence" (RRS) in this study; this is a 10-nucleotide sequence containing the conventional SD sequence, as illustrated in Figure 1A. Although the consensus SD sequence is generally known to be critical for ribosomal recruitment, the ribosome-binding affinity is truly mediated by a longer (10-nucleotide) region that includes the SD sequence and can hybridize with a sequence at the 3' end of the 16S rRNA [19,24].

In summary, we can obtain the probability of a given mRNA being bound to a ribosome using kinetic equations derived from: (1) the probability of each conformation; (2) the exposure probability of the RDS for each conformation; and (3) the hybridization energy of the RRS and anti-RRS. The probability of ribosome-bound mRNA enables us to estimate the translational efficiency since ribosome-bound mRNAs produce proteins and also to design mRNA sequences with desired expression levels.

### Global folding of transcribed mRNAs

As shown in Figure 1B (Equation 1), transcribed mRNAs fold into diverse conformations according to their structural energies [36,37]. We used the *UNAFold *v3.3 secondary structure-predicting software to estimate the Gibbs free energy of each conformation [38,39]. The probability of a given mRNA molecule existing in a given conformation is obtained using a partition function for the predicted conformations and their Gibbs free energies [34], as follows:(1)

where *T*_*mRNA *_denotes the total number of mRNAs that may be transcribed from the gene of interest, *S*_*i *_denotes one of the conformations of the transcribed mRNAs, Δ*G*(*S*_*i*_) denotes the Gibbs free energy of *S*_*i*_, *R *denotes the gas constant, *T *denotes an absolute temperature, and *l *denotes the number of predicted conformations.

### Regional unfolding at the ribosome-docking site

We define the RDS as the 30-nucleotide sequence starting from the first nucleotide of the RRS (Figure 1A), as this has been structurally identified as the region where a ribosome actually sits on an mRNA molecule [18,30-32]. The RDS spans the RRS and several N-terminal codons. Thus, in order to determine an RDS, we must identify the RRS (see *Ribosome binding *below).

Although ribosomes and elongation factors can cooperate to unwind helical structures during translation elongation, ribosomes cannot unwind base-paired mRNA structures during translation initiation [27,28,40]. Thus, in order for a ribosome to bind, the RDS must lose (through unfolding) any secondary structure that might prevent ribosomal docking. For example, the mRNA of the MS2 phage replicase gene is not translated unless the secondary structure around the start codon is disrupted [41,42]. Due to the inability of helical mRNA structures to be unwound during translation initiation, we herein modeled the probability that all secondary structures in an RDS would be unfolded at any given moment. This is called the "exposure probability."

The exposure probability of each conformation is summed to obtain an overall exposure probability for a given RDS (Equation 2). As the probability of each conformation differs according to its stability (Equation 1), the exposure probability of a certain conformation is multiplied by the probability of the corresponding conformation to obtain the overall RDS-exposure probability.(2)

In Equation 2, *p*(*S*_*i*_) denotes the probability of the *i*-th conformation *S*_*i*_, *p*_*i *_denotes the RDS-exposure probability of the *i*-th conformation *S*_*i*_, and *P*_*ex *_denotes the overall RDS-exposure probability.(3)(4)

In Equations 3 and 4, *θ*_*i,j *_denotes the nucleotide-unpairing probability of the *j*-th nucleotide in an RDS of conformation *S*_*i*_, Δ*G*_*i,j *_denotes the Gibbs free energy of a stack structure in a ribosome-docking site containing the *j*-th nucleotide, and *L *denotes the number of nucleotides in a given stack structure.

The nucleotides in an RDS may be either paired or unpaired. The ribosome-docking site-exposure probability (*p*_*i*_) of a given conformation *S*_*i *_is defined as the product of the unpairing probabilities for all nucleotides in the site (Equation 3).

The nucleotides in a loop structure are free of base pairing. If a nucleotide is in a loop region, no Gibbs free energy term is required, and its unpairing probability is 1. In contrast, the nucleotides in a stack structure are base paired, and the ability of the nucleotides in a stack structure to lose their base pairings depends on the structural flexibility of the stack. Their unpairing probabilities are calculated using a partition function similar to that shown in Equation 1; here, it is assumed that all nucleotides in the stack must simultaneously lose their base pairings in order for the stack structure to unfold (Equation 4). More specifically, a stack structure has only two possible states: folded and unfolded. Therefore, the probability that a stack is unfolded is 1/(1+exp(-Δ*G*_*stack*_/*RT*)), where the Gibbs free energy of an unfolded stack is 0 kcal/mol. As all of the nucleotides in a stack must lose their base pairings simultaneously in order for a stack to unfold, the product of all of the nucleotide-unpairing probabilities should equal the stack-unfolding probability. For instance, if a stack structure consisting of four nucleotides has an unfolding probability of 0.0001, then the nucleotide-unpairing probabilities of the four nucleotides are equal to 0.1 ().

### Ribosome binding

#### Ribosome-recognizing sequence identification

Once an RDS is unfolded and exposed, ribosome binding is mediated by hybridization of the RRS in the RDS with an anti-RRS sequence present in the ribosomal 16S rRNA (Figure 1B) [18]. Thus, the RRS must be identified not only to allow definition of the RDS (see above), but also to allow us to calculate the hybridization affinity between the RRS and anti-RRS.

Approximately 10 nucleotides at the 3' end of the 16S rRNA are involved in the hybridization of a ribosome with an mRNA sequence [19,24]. We therefore defined the RRS as a 10-nucleotide sequence complementary to the anti-RRS sequence in the 3' end of 16S rRNA. In the case of *E. coli*, the anti-RRS sequence is 5'-*UCACCUCCUU*-3' [33], and the RRS contains a variation of the consensus SD sequence [43], which is capable of hybridizing with part of the anti-RRS.

In order to identify an RRS within a given mRNA sequence, we computationally hybridized every 10-nucleotide sequence in the region from -30 to -10 upstream of the start codon to the anti-RRS sequence, and estimated the hybridization energy using the hybrid-2s program contained within the *UNAFold *software package [38]. We then selected one of the lowest-energy sequences for use as the RRS [24,44].

#### Ribosome-binding kinetics

In order to estimate the probability of ribosome-bound mRNA, we modeled ribosome binding using ordinary differential equations (Equation 5). An RDS folds or unfolds thermodynamically, and an unfolded RDS can recruit free ribosomes according to the hybridization affinity between the RRS and anti-RRS.

Equation 5 does not include the RDS folding reaction, as the number of folded or unfolded RDSs can be obtained from the RDS's overall exposure probability. Here, we assume that the RDS-folding reaction is relatively faster than ribosome binding, and thus reaches equilibrium.(5)

In Equations 5 and 6, *m*_*E*_, *m*_*R *_and *R*_*F *_denote the number of RDS-exposed mRNAs, ribosome-bound mRNAs, and free ribosomes, respectively; *k*_*f *_and *k*_*r *_denote the ribosome-association and -dissociation rate constants, respectively; and *s *denotes the number of bound ribosomes (polysomes) per transcript.(6)

The probability of a given mRNA being bound to a ribosome (*P*_*c*_) at steady state is then derived (Equation 7) from Equations 5 and 6. Here, the ribosome-association and -dissociation reaction constants, *k*_*f *_and *k*_*r*_, are replaced with an equilibrium constant (*K*_*R*_) that is obtained from the hybridization energy between the RRS and anti-RRS (*ΔG*_*R*_) (Equation 6), as estimated using the hybrid-2s program in the *UNAFold *software package [38]. The numbers of RDS-exposed and ribosome-bound mRNAs are replaced with the total number of mRNA molecules, which is calculated as *T*_*mRNA *_= *m*_*E*_/*P*_*ex*_+*m*_*R*_.(7)

In , *R*_*T *_denotes the total number of ribosomes in a cell (*R*_*T *_= *R*_*F *_+ *s*·*m*_*R*_), *K*_*R *_denotes an equilibrium constant derived from the hybridization energy (*ΔG*_*R*_), *P*_*ex *_denotes an overall RDS-exposure probability, and *P*_*C *_denotes the probability of ribosome-bound mRNA.

As shown in Equation 7, three parameters are needed to calculate the probability of ribosome-bound mRNA: the total numbers of mRNAs in the cell (*T*_*mRNA*_), the total number of ribosomes in the cell (*R*_*T*_), and the number of ribosomes per polysome (*s*). We obtained the necessary parameters from the literature. For the *luxR *gene used as an example below, the total number of *luxR *transcripts was calculated using a kinetic equation for transcription at steady state: [transcribed mRNA] = [gene copy number] × [transcription rate] × [mRNA half-life]/ln(2). For the utilized plasmid, the *luxR *gene is transcribed at a rate of 20 mRNAs/min under the control of the *lac *promoter, and there are about 100 copies of the gene per cell [45]. The half-life of the *luxR *mRNA is assumed to be about 2 min, as this is the average half-life of an mRNA in *E. coli *[46,47]. Therefore, the total number of *luxR *mRNAs (*T*_*mRNA*_) is taken to be approximately 5,700 per cell. The number of ribosomes in a given *E. coli *cell (*R*_*T*_) is about 57,000 [48], and the number of ribosomes simultaneously translating a given transcript (*s*) is 20 [49].

### Translational efficiency

We herein define the probability of a given mRNA being bound to a ribosome as translational efficiency, since protein production is proportional to the number of bound ribosomes [23]. However, it has been reported that an RRS with a strong hybridization energy (*i.e*., lower than -13 kcal/mol) could have a 10-fold lower translational efficiency [28,50,51]. Therefore, when a given RRS had a strong hybridization energy, we decreased the transcript's translational efficiency by a factor of 0.1.

### Estimation of translational efficiency: an example

**Figure 2:**
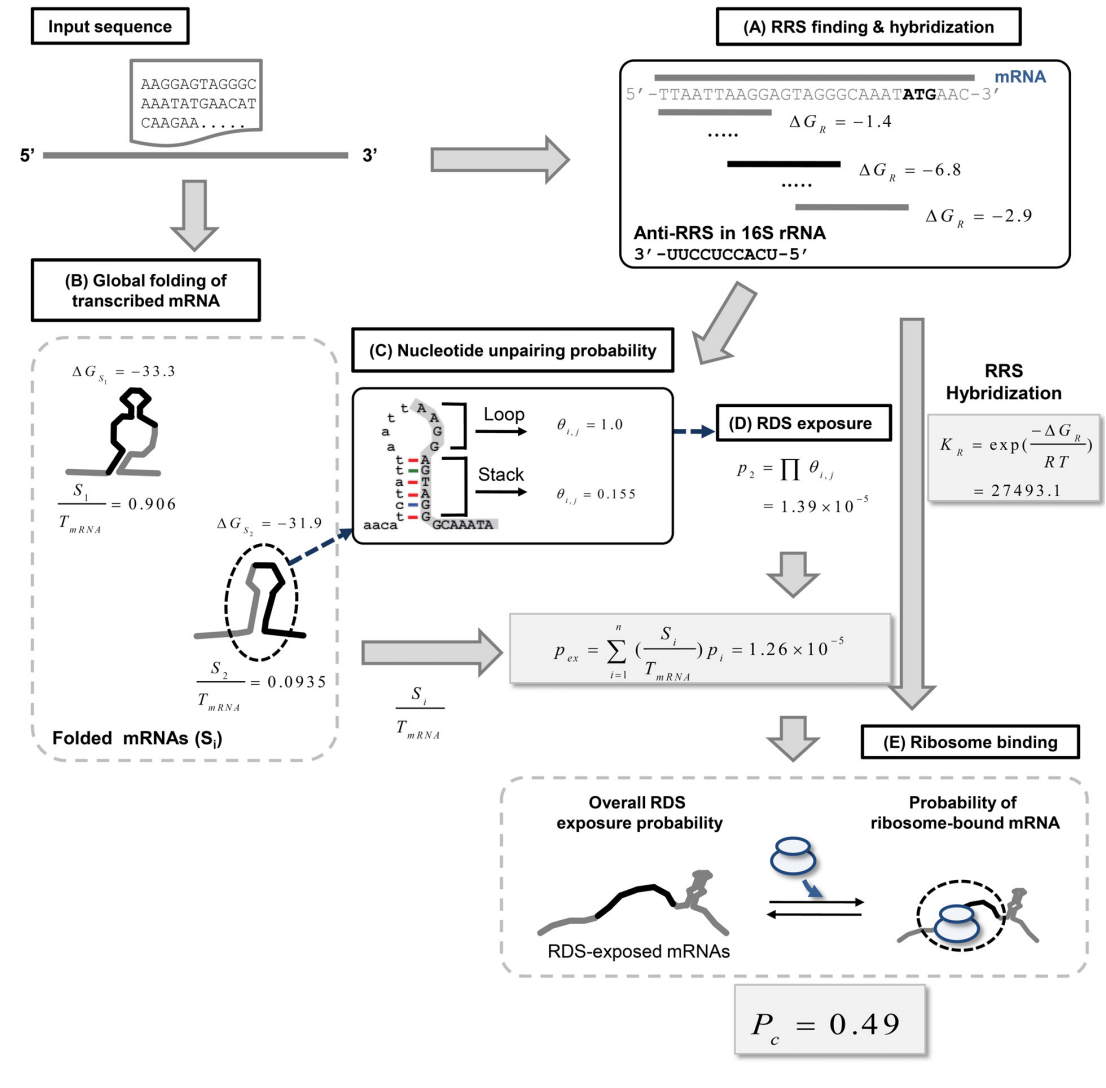
**An example of estimating translational efficiency using our model**.

Here we show an example of our model's translational efficiency estimation (Figure 2). First, we identified an RRS upstream of the start codon. We used this RRS to determine the RDS, calculate the overall exposure probability of the RDS, and compute the probability of ribosome-bound mRNA. As shown in Figure 2A, among the various 10-nucleotide sequences from the mRNA, *AAGGAGTAGG *was found to have the lowest hybridization energy (*ΔG*_*R *_= -6.8 kcal/mol) to the anti-RRS sequence, and was thus selected as the RRS.

Second, we predicted the possible secondary structures of the mRNA sequence. In the example shown, the mRNA may fold into two different conformations with Gibbs free energies of -33.3 and -31.9 kcal/mol (Figure 2B). The probability for each conformation was obtained from the free energies using Equation 1. The results revealed that the conformation with the free energy of -33.3 kcal/mol comprised 90.6% of all folded versions of this mRNA, while the other conformation (free energy, -31.9 kcal/mol) comprised only 9.4%.

Third, we calculated an overall RDS-exposure probability from the RDS-exposure probability of each conformation. Each RDS-exposure probability was calculated from the nucleotide-unpairing probabilities of the nucleotides in that RDS. As shown in Figure 2C, the RDS (upper-case letters) of the conformation with the Gibbs free energy of -31.9 kcal/mol had a stem-loop structure composed of one loop and one stack. The nucleotide-unpairing probabilities in the loop region were taken as 1, while those in the stack were calculated using Equation 4:

Consequently, the RDS-exposure probability of the second conformation was the product of all unpairing-probabilities for the nucleotides in the RDS (Figure 2D), as follows:

If the mRNA sequence of interest can fold into more than two different conformations, the same calculation would be carried out for each conformation. The RDS-exposure probability of the first conformation in the example was found to be *p*_*1 *_= 9.26 ×10^-9^. The overall RDS-exposure probability was the sum of the two RDS-exposure probabilities (*p*_*1 *_and *p*_*2*_), multiplied by each conformation probability, as follows:

Fourth, we calculated the probability of ribosome-bound mRNA using Equation 7. For this calculation, we used parameter values obtained from the literature: There are 57,000 ribosomes in a cell (*R*_*T *_= 57,000) [48]; 20 ribosomes simultaneously produce proteins from a given mRNA sequence in the form of a polysome (*s *= 20) [49]; and there are 5,700 transcribed mRNAs in total (*T*_*mRNA *_= 5,700) [45-47]. The equilibrium constant (*K*_*R*_) for ribosome association and dissociation was derived using Equation 6, using the hybridization energy of the identified RRS (*ΔG*_*R *_= -6.8 kcal/mol) with the anti-RRS sequence (Figure 2A).

As shown in Figure 2E, the probability of a given mRNA being bound to a ribosome was *P*_*C *_= 0.49, indicating that about 49% of the transcribed mRNA sequences were occupied by ribosomes (and were therefore producing proteins). According to our definition, this corresponds to the translational efficiency of *E *= 0.49.

### Computational design of synthetic ribosome-docking site sequences

To design mRNA sequences that should be expressed at the desired protein levels, we created 22 synthetic RDS sequences with diverse translational efficiencies ranging from 10^-5 ^to 1. Although the RDS as defined herein contains several N-terminal codons, we only mutated nucleotides upstream of the start codon, so as to avoid altering any protein properties.

**Figure 3:**
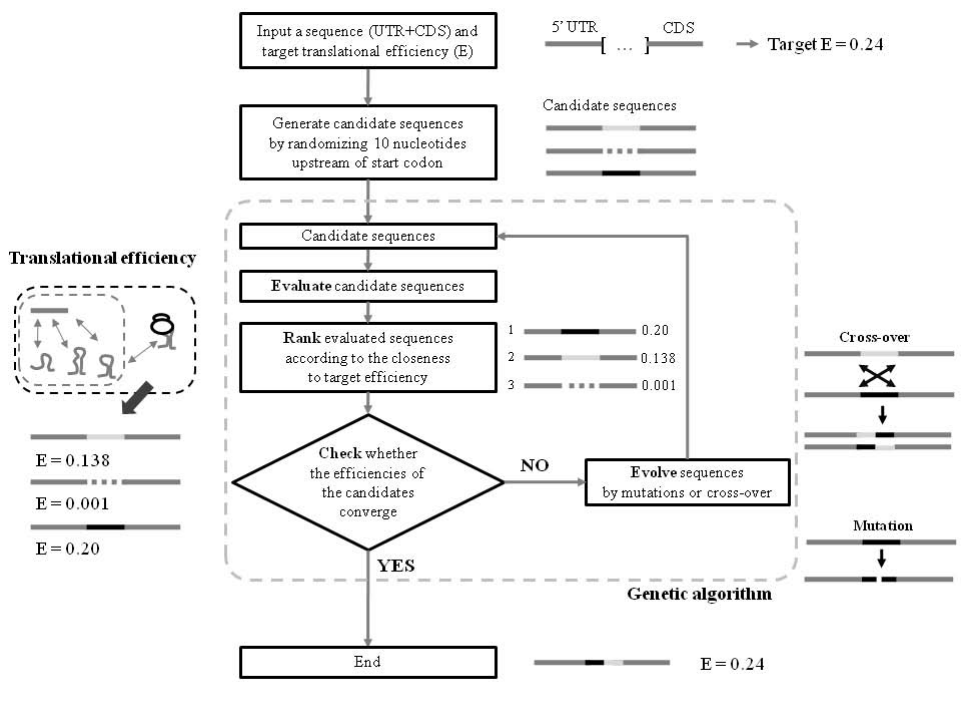
**The use of a genetic algorithm to design synthetic RDS sequences having specific translational efficiencies**. The RDS design process starts with a user-specified 5' untranslated region (UTR) and a coding sequence (CDS). The 10 nucleotides upstream of the start codon, which make up part of the RDS, are modified to satisfy a target translational efficiency using a genetic algorithm. The generated sequences are randomly mutated or crossed over. If the translational efficiency of the generated sequences converges to the target efficiency, the algorithm terminates.

As shown in Figure 3, a genetic algorithm was used to computationally optimize the 10 nucleotides upstream of the start codon, with the goal of designing an mRNA sequence with a specific translational efficiency. Briefly, the 10 nucleotides upstream of the start codon were randomized to generate 100 different mRNA sequences, and the translational efficiency of each mRNA was estimated and ranked relative to the target translational efficiency. The 10 highest-ranked sequences proceeded to the next round of the algorithm without modification, while 90 new mRNA sequences were generated by crossing-over or mutating randomly selected sequences from the 100 original mRNA sequences. The translational efficiencies of the newly generated sequences were then estimated, ranked, and selected as described above. This process was repeated until the translational efficiency of the best-fit mRNA sequence converged to the target translational efficiency.

### Plasmid construction

For cloning of *luxR *gene sequences harboring diverse synthetic RDS sequences under the control of the *lac *promoter, we constructed a customized vector containing the RSF replication origin and *lacI*^*q *^gene. The RSF replication origin and *lacI*^*q *^were cloned from pRSF-Duet (Novagen). The *lac *promoter (*P*_*lac*_) and *lacZα *gene were cloned from pBluescript (Stratagene). The kanamycin resistance gene was cloned from pCR-Blunt II-TOPO (Invitrogen). The 5' coding sequence of *luxR *from *Vibrio fischeri *(ATCC 700601D) was first modified based on codon degeneracy in order to increase the GC content, and then PCR was used to create and amplify *luxR *genes with various synthetic RDS sequences. The gene encoding DsRed2 was cloned from pDsRed2-N1 (Clontech). The utilized primers are described in *Additional file *1.

### Measurement of *luxR *expression

The generated *luxR *genes were fused with *lacZα *as described above, and their expression levels were measured by β-galactosidase assays, as reported previously [52,53]. In brief, *E. coli *DH5α cells were transformed with the constructed plasmids and cultured to log phase in LB broth containing 0.1% glucose. The cells were induced with 2.5 mM isopropyl β-D-1-thiogalactopyranoside (IPTG) for 2 hours, and then harvested and resuspended in Z-buffer (4.27 g Na_2_HPO_4_, 2.75 g NaH_2_PO_4_H_2_O, 0.375 g KCl, 0.125 g MgSO_4 _7H_2_O, and 1.4 ml β-mercaptoethanol in 500 ml distilled water). Resuspended cells (150 μl) were permeabilized with 5 μl of 0.1% SDS and chloroform. After equilibration for 2 min, 60 μl of o-nitrophenyl-β-D-galactoside was added. The cells were then incubated until yellow color developed, at which point 150 μl 1 M Na_2_CO_3 _was added. The samples were centrifuged at 13,000 rpm for 1 min for removal of cell debris, and the optical density at 420 nm (OD_420_) was measured. The activity of β-galactosidase was calculated as follows:

### Measurement of DsRed2 expression

*E. coli *DH5α cells harboring a DsRed2 plasmid were cultured to stationary phase, and red fluorescence intensity was measured using a Tecan Infinite M200 instrument (excitation at 558 nm and emission at 583 nm). The results were normalized with respect to the optical density at 600 nm (OD_600_).

## Results and Discussion

### Validation using data in the literature: the MS2 coat protein gene

**Figure 4:**
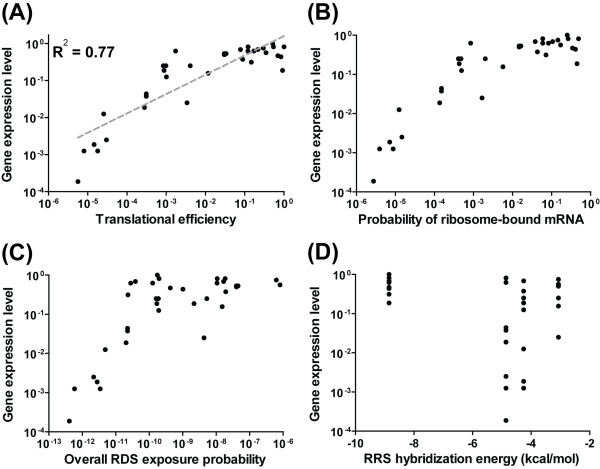
**The estimated translational efficiencies of the modified RDS sequences for the MS2 coat gene**. The estimated translational efficiencies (A), probabilities of ribosome-bound mRNA (B), overall RDS-exposure probabilities (C), RRS-hybridization energies (D), and expression levels of MS2 coat genes harboring modified RDS sequences as well. As the expression levels were normalized, the estimated translational efficiencies were also normalized for comparison. The estimated translational efficiency had a high correlation (*R*^2 ^= 0.77) to the gene expression level when linearly regressed. Consistent with the experimental study, the properties of the MS2 coat genes were estimated at 42°C.

We confirmed the validity of our model using expression data for constructs in which the MS2 coat gene was ligated to diverse RDS sequences shown to yield various RDS secondary structures and ribosome-binding affinities [33]. We used our model to predict the translational efficiencies, ribosome-bound mRNA probabilities, overall RDS-exposure probabilities, and RRS-hybridization energies for the various MS2 coat gene mRNA sequences. The highest MS2 coat protein expression level achieved in the experiments had been normalized to 1 in the previous study, so we normalized our highest ribosome-bound mRNA probability to 1 for comparison. Our model successfully predicted the relative expression levels, with a high correlation of *R*^*2 *^= 0.77 (Figure 4A). The other estimated properties are shown in Figure 4.

Interestingly, a sequence showing a relative expression level of 0.44 was estimated to have an overall RDS exposure probability of 1.0 × 10^-9 ^and a ribosome-bound mRNA probability of 0.41. In other words, only 1 out of RDS regions was predicted to be naturally exposed to ribosomes, but 41% of the overall RDS regions were predicted to be occupied by ribosomes. Our model estimated that despite the severely low RDS-exposure probability, the RDS had a strong ribosome-binding affinity (hybridization energy, *ΔG *= - 8.9 kcal/mol). Therefore our model suggests that once a ribosome was bound to this sequence it would rarely detach from the RDS, while unbound RDS regions would be dynamically driven to the unfolded state in order to resolve their disequilibrium.

### Computational design of *luxR *mRNA sequences with desired translational efficiencies

**Figure 5:**
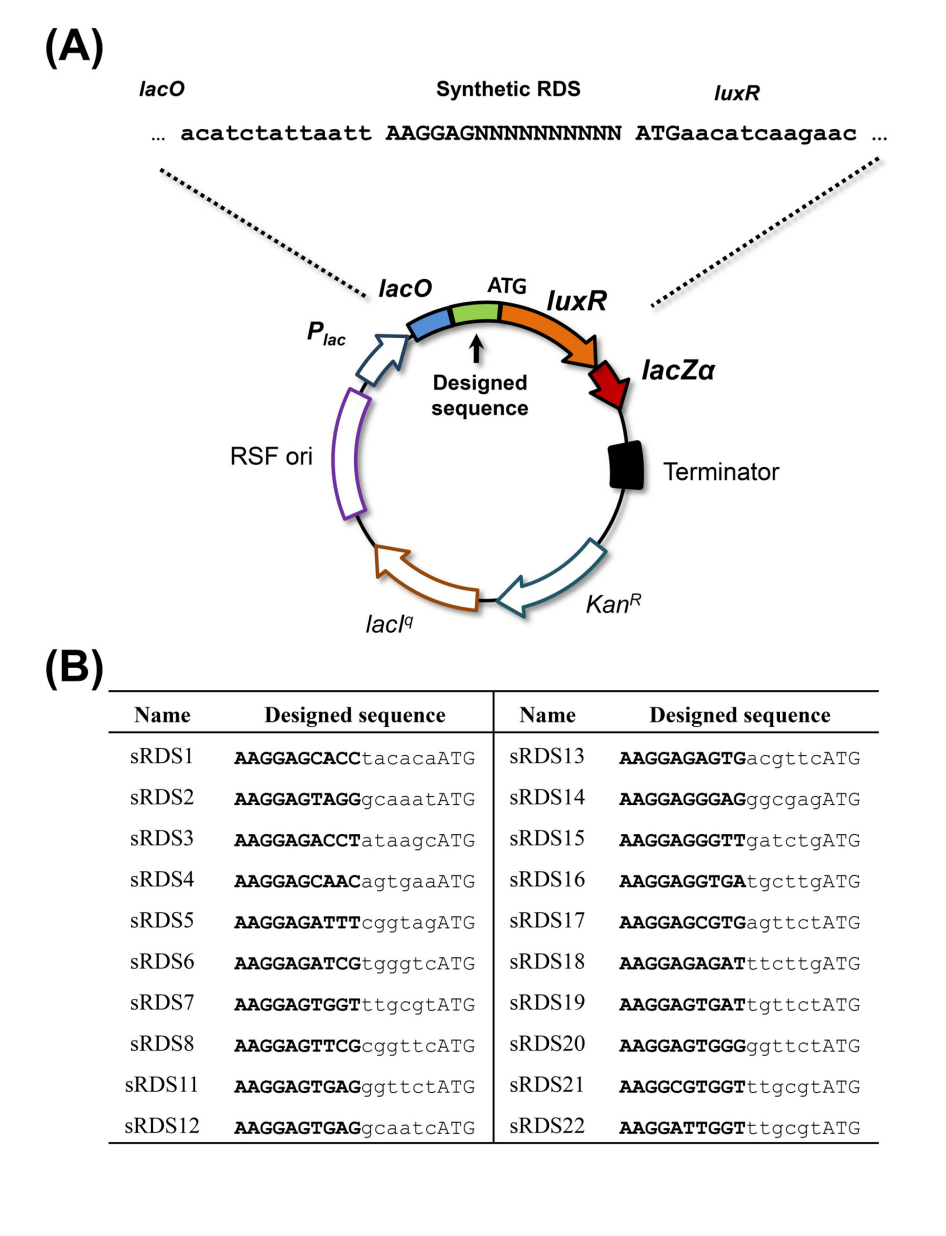
**The constructed plasmids and designed synthetic RDS sequences**. (A) Schematic of the plasmid harboring the *luxR *gene with the designed RDS sequences. Based on a genetic algorithm, the 10 nucleotides upstream of the start codon were modified to create synthetic RDS sequences with specific translational efficiencies. For measurement of LuxR protein expression, the *lacZα *gene was fused to the *luxR *gene. (B) The designed synthetic RDS sequences are listed; the RRS sequences are indicated in bold. For sRDS21 and 22 they have the same designed spacer sequence but their SD sequences were modified further.

To experimentally validate our model, we chose the *luxR *gene from *V. fischeri *for mRNA sequence design. We computationally generated 22 synthetic mRNA sequences, specifically RDS sequences, predicted to have various translational efficiencies (ranging from 10^-5 ^to 1; Figure 3). The plasmid structure is shown in Figure 5A and the designed synthetic RDS sequences are listed in Figure 5B.

**Figure 6:**
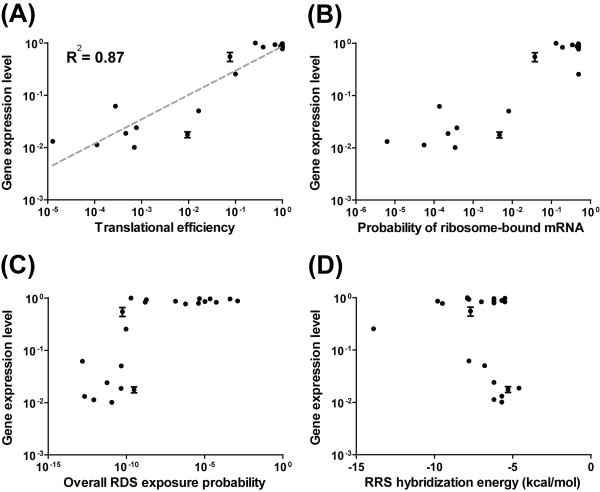
**The estimated properties of the synthetic *luxR *mRNA sequences**. The properties of the synthetic RDS sequences, including their translational efficiencies (A), probabilities of ribosome-bound mRNA (B), overall RDS-exposure probabilities (C), and RRS-hybridization energies (D), were estimated and compared with normalized *luxR *expression levels averaged from three independent β-galactosidase assays. The translational efficiencies of the designed sequences were normalized for comparison.

The resulting *luxR *expression levels are shown in Figure 6, along with the estimated properties of the synthetic RDS sequences, including the translational efficiency, the probability of ribosome-bound mRNA, the overall RDS-exposure probability, and the RRS-hybridization energy for each sequence. The *luxR *expression levels observed in our experiments were consistent with our design goals, showing a strong correlation of *R*^*2 *^= 0.87.

**Figure 7:**
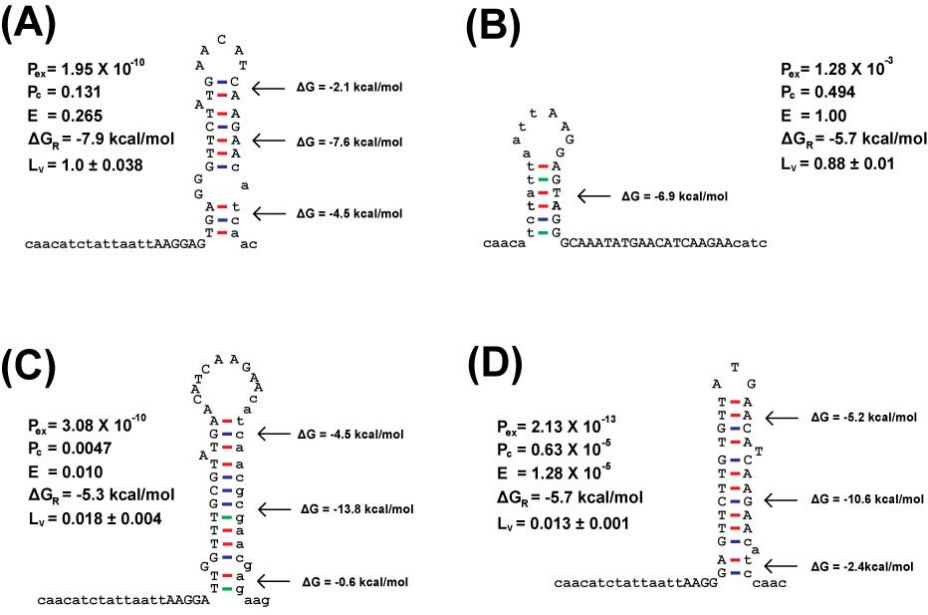
**The predicted secondary structures and estimated properties of high-and low-expressing synthetic RDS sequences**. The RDS nucleotides considered in this calculation are shown in upper-case letters, and the estimated translational efficiencies (*E*), measured relative expression levels (*L*_*v*_), overall RDS exposure probabilities (*P*_*ex*_), probability of ribosome-bound mRNA (*P*_*C*_), RRS-anti RRS hybridization energies (*ΔG*_*R*_) and the Gibbs free energies (Δ*G*) of the helix structure inside RDS are also shown for sRDS11 (A), sRDS2 (B), sRDS22 (C) and sRDS9 (D). The secondary structures were predicted by *UNAFold*.

Among the synthetic sequences, the overall RDS-exposure probabilities and RRS-hybridization energies of the high-expressing RDS sequences varied from 10^-10 ^to 10^-3 ^and from -9.8 to -5.5 kcal/mol, respectively, while those of the low-expressing sequences varied from 10^-13 ^to 10^-10 ^and -13.9 to -4.6 kcal/mol, respectively. As shown in Figures 7A and 7B, the high-expressing sequences had high exposure probabilities caused by the presence of many unpaired nucleotides in the RDS region, and they had strong RRS-hybridization energies. In contrast, the low-expressing sequences had both low exposure probabilities and low hybridization energies (Figure 7C and 7D). Our experimental results revealed that the LuxR protein expression levels among the high- and low-expressing sequences were consistent with the estimated hybridization energies and exposure probabilities: A high exposure probability and hybridization energy enhanced ribosome binding and thereby increased protein expression, while a low exposure probability and hybridization energy prevented ribosome binding and thereby decreasing protein expression.

For example, sRDS11 and sRDS22 had similar exposure probabilities (10^-10^), but the hybridization energy of sRDS11 was stronger by -2.6 kcal/mol, increasing the ribosome-binding equilibrium constant by about 70-fold (Figures 7A and 7C). The estimated translational efficiencies of sRDS11 and sRDS22 were 0.265 and 0.010, respectively, and their expression levels were 1 and 0.020, respectively. As another example, sRDS9 and sRDS2 had the same hybridization energies, but sRDS9 had a lower exposure probability, showed a 70-fold lower expression level, and was estimated to have a translational efficiency of 1.28 × 10^-5 ^due to its extremely low exposure probability. The structures and estimated properties of sRDS2 and sRDS9 are shown in Figures 7B and 7D, respectively.

### Application of the model to protein over-production: over-expression of DsRed2

**Figure 8:**
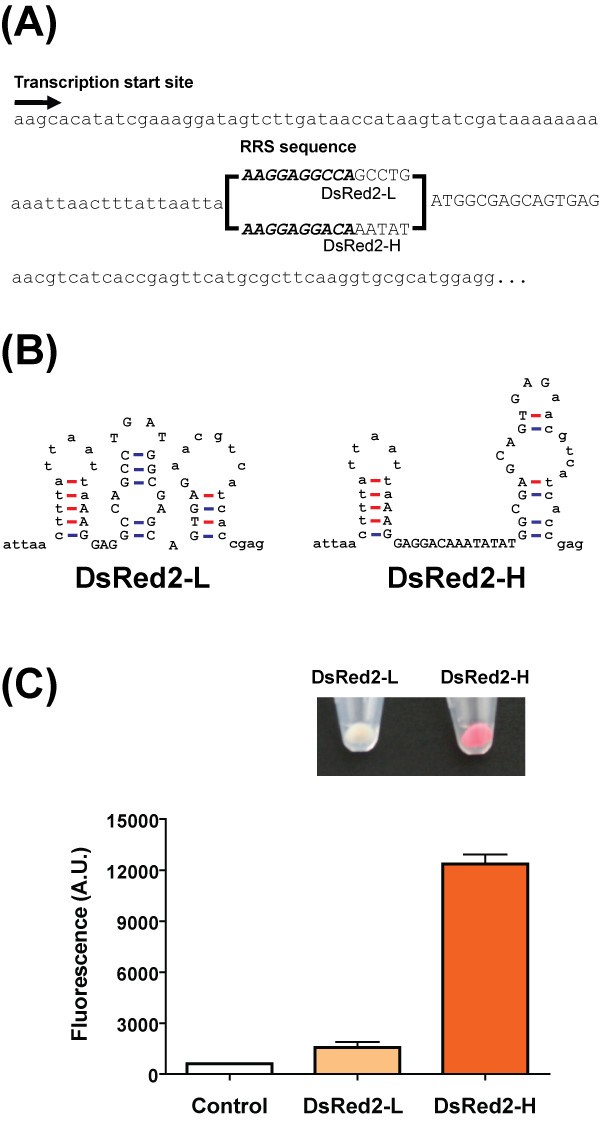
**The designed DsRed2 transcripts and measurement of red fluorescence intensities**. (A) The transcript sequences, including the designed RDS nucleotides, of the DsRed2 gene. The RRS sequences are in bold and the RDS regions are in upper-case letters. (B) The predicted RDS secondary structures of the designed DsRed2 mRNA sequences. The RDS nucleotides are in upper-case letters. (C) The expression levels of the designed DsRed2 mRNAs in control (null plasmid), DsRed2-L (designed for low expression), and DsRed2-H (designed for over-expression) experiments. The cells containing the designed DsRed2 genes are shown over the plot.

We applied our model to the issue of over-expressing the DsRed2 gene. This gene is not expressed well in plasmids other than its original vector, due to the presence of RDS secondary structures that severely block ribosome binding [7]. We designed a DsRed2 transcript sequence predicted to have a high translational efficiency (DsRed2-H) for over-expression, and one with a low translational efficiency (DsRed2-L) for comparison. The translational efficiencies of the designed transcripts were 0.49 for DsRed2-H and 0.072 for DsRed2-L. The designed mRNA sequences, predicted RDS secondary structures, and resulting expression levels are shown in Figure 8.

Our results revealed that modification of only eight nucleotides upstream of the start codon dramatically changed the secondary structure of the RDS region, resulting in an approximately 10-fold increase in protein expression compared to that of DsRed2-L (Figure 8). This increased expression resulted from a large change in the RDS-exposure probability: DsRed2-H had an overall RDS-exposure probability of 2.12 × 10^-5^, whereas the exposure probability of DsRed2-L was six orders of magnitude lower, at 2.78 × 10^-11^, due to differences in the stem-loop structures (Figure 8B). Although DsRed2-H could form a more stable mRNA-ribosome complex than DsRed2-L, the difference in hybridization energy did not significantly change ribosome recruitment compared with the change in the overall RDS-exposure probability. The RRS-hybridization energies of DsRed2-H and DsRed2-L were -9.3 kcal/mol and -8.6 kcal/mol, respectively. Consequently, our model suggests that the variation in RDS secondary structure induced by our model-directed mutations caused a significant change in DsRed2 protein expression.

## Conclusions

We herein describe a mathematical model for estimating mRNA translational efficiency based on the effect of RDS secondary structures and the RRS-hybridization events that occur during translation initiation. We confirmed the validity of our model using previously reported expression data, and further confirmed our model experimentally using computationally designed synthetic RDS sequences ligated to *luxR*. The experimentally determined gene expression levels of the synthetic RDS sequences were consistent with the efficiencies targeted by our model; the correlation coefficient (*R*^*2*^) was 0.87 when linearly regressed.

Salis *et al. *recently proposed a thermodynamic model for estimating the translational efficiency of mRNA sequences [54]. Their model predicts the relationship between the protein expression level and the summed Gibbs free energies, including the hybridization energy of the SD sequence and the 16S rRNA, the energy of helical structures within the ribosome binding site, and so on. Unlike the previously proposed model, our model is a steady-state kinetic model based on the stepwise process of translation initiation, from the global folding of transcribed mRNA to ribosome binding. Therefore, our system precisely models the translation-initiation process based on the reactions involved in initiation, not through a simple summation of energy terms. Although these two approaches differ to some extent, the predictive value of both models is similar, since the same key factors (the secondary structure around the start codon and the ribosome-binding affinity) are taken as determining translation efficiency. However, the incorporation of actual translation processes into our model gives us detailed information on the translation-initiation process. For example, we can assess how much each global mRNA structure affects the translation efficiency, how many ribosome-docking sites within the transcribed mRNAs are exposed to ribosomes, and how many mRNAs are occupied by ribosomes.

Furthermore, since we precisely model the stepwise processes involved in translation without any parameter fittings, our model could be extended to the examination of other biological processes related to translation initiation. For example, the incorporation of a molecule capable of competing with ribosomes would allow us to model the process of translation repression by small regulatory RNAs, which inhibit translation initiation by competing with ribosomes [55]. In addition, since our model assumes that transcribed mRNAs may fold into two or more potential structures depending on their structural Gibbs free energies, the model could be used to predict the translation efficiencies of mRNA sequences that have two or more folded structures at equilibrium [56].

Since our model is capable of estimating the translational efficiency of mRNA sequences, it can be utilized to facilitate the assembly of genetic elements into robust synthetic cellular systems. In natural systems, the kinetics of the various elements has been evolutionarily optimized for robust operation. Similarly, we must optimize the kinetics of assembled elements in designed systems; however, such optimization has proven to be a main challenge hampering the development of robust synthetic systems. Our model can therefore assist in the design of synthetic RDS sequences that will help researchers obtain mRNA sequences with translation rates that will best fit the kinetics of their designed systems. Finally, our model can also be utilized to design optimal mRNA sequences for over-expressing proteins of interest. This could be especially useful for the production of therapeutic human proteins such as interleukin-10, which is weakly expressed in bacterial cells due to the presence of strongly folded RDS structures [12]. In combination with such engineering, protein expression levels could be synergistically elevated through the use of conventional translation elongation-optimization methods, such as codon optimization.

## Authors' contributions

DN developed a translation initiation model to estimate translational efficiency, and designed the DNA sequences for the experiments. SL gave valuable comments during model construction, and conducted the validation experiments. DL was involved in model construction and the drafting of the manuscript. All authors drafted, read and approved the final manuscript.

## Supplementary Material

Additional file 1 **List of the primers used in our experiments**. The primers used to construct the expression vectors, as well as the synthetic RDS-containing mRNA sequences, are listed in this file. Click here for file
