# Supplementary material for: Mathematical modeling of translation initiation for the estimation of its efficiency to computationally design mRNA sequences with desired expression levels in prokaryotes
Source: BMC Syst Biol. 2010 May 26;4:71. doi: 10.1186/1752-0509-4-71 (PMC2883959; doi:10.1186/1752-0509-4-71)
Supplement: Additional file 1 — List of the primers used in our experiments. The primers used to construct the expression vectors, as well as the synthetic RDS-containing mRNA sequences, are listed in this file. [file 1752-0509-4-71-S1.DOC]

Table 1. List of primers used in our experiments

| Primer | Orientation | Sequence(5’-3’) ^a^ |
| --- | --- | --- |
| pLac | Forward | 5’ ggtacccaatacgcaaaccgcctctcc |
|  | Reverse | 5’ ttaattaatagatgttgtgtgaaattgttatccgctcaca |
| *luxR*-F1 | Forward | 5’ aacaacaaggacatcaaccagtgcttgtcggagatagcaaagataatacattgtgaatattacc  tattcgctattatctatcctcactc |
| *luxR* -F2 | Forward | 5’ atgaacatcaagaacatcaacgcgaacgagaagatcatcgacaacatcaagacgtgcaacaaca  acaaggacatcaaccagtgct |
| *luxR* -F3 | Forward | 5’ atgaacatcaagaacatcaacgcgaacgag |
| *luxR* -R | Reverse | 5’ gaattcttaatttttaaggtatggacaattaatggcg |
| sRDS1-F | Forward | 5’ ttaatt**AAGGAGCACC**tacaca**ATG**aacatcaagaacatcaacgc |
| sRDS2-F | Forward | 5’ ttaatt**AAGGAGTAGG**gcaaat**ATG**aacatcaagaacatcaacgc |
| sRDS3-F | Forward | 5’ ttaatt**AAGGAGACCT**ataagc**ATG**aacatcaagaacatcaacgc |
| sRDS4-F | Forward | 5’ ttaatt**AAGGAGCAAC**agtgaa**ATG**aacatcaagaacatcaacgc |
| sRDS5-F | Forward | 5’ ttaatt**AAGGAGATTT**cggtag**ATG**aacatcaagaacatcaacgc |
| sRDS6-F | Forward | 5’ ttaatt**AAGGAGATCG**tgggtc**ATG**aacatcaagaacatcaacgc |
| sRDS7-F | Forward | 5’ ttaatt**AAGGAGTGGT**ttgcgt**ATG**aacatcaagaacatcaacgc |
| sRDS8-F | Forward | 5’ ttaatt**AAGGAGTTCG**cggttc**ATG**aacatcaagaacatcaacgc |
| sRDS9-F | Forward | 5’ ttaatt**AAGGAGTTCT**tgtgtt**ATG**aacatcaagaacatcaacgc |
| sRDS10-F | Forward | 5’ ttaatt**AAGGAGAGAG**ttcttg**ATG**aacatcaagaacatcaacgc |
| sRDS11-F | Forward | 5’ ttaatt**AAGGAGTGAG**ggttct**ATG**aacatcaagaacatcaacgc |
| sRDS12-F | Forward | 5’ ttaatt**AAGGAGTGAG**gcaatc**ATG**aacatcaagaacatcaacgc |
| sRDS13-F | Forward | 5’ ttaatt**AAGGAGAGTG**acgttc**ATG**aacatcaagaacatcaacgc |
| sRDS14-F | Forward | 5’ ttaatt**AAGGAGGGAG**ggcgag**ATG**aacatcaagaacatcaacgc |
| sRDS15-F | Forward | 5’ ttaatt**AAGGAGGGTT**gatctg**ATG**aacatcaagaacatcaacgc |
| sRDS16-F | Forward | 5’ ttaatt**AAGGAGGTGA**tgcttg**ATG**aacatcaagaacatcaacgc |
| sRDS17-F | Forward | 5’ ttaatt**AAGGAGCGTG**agttct**ATG**aacatcaagaacatcaacgc |
| sRDS18-F | Forward | 5’ ttaatt**AAGGAGAGAT**ttcttg**ATG**aacatcaagaacatcaacgc |
| sRDS19-F | Forward | 5’ ttaatt**AAGGAGTGAT**tgttct**ATG**aacatcaagaacatcaacgc |
| sRDS20-F | Forward | 5’ ttaatt**AAGGAGTGGG**ggttct**ATG**aacatcaagaacatcaacgc |
| sRDS21-F | Forward | 5’ ttaatt**AAGGCGTGGT**ttgcgt**ATG**aacatcaagaacatcaacgc |
| sRDS22-F | Forward | 5’ ttaatt**AAGGATTGGT**ttgcgt**ATG**aacatcaagaacatcaacgc |
| sRDS-R | Reverse | 5’ gcggccgcatttttaaggtatggacaattaatggcgc |
| LacZα | Forward | 5’ cgcggccgctggtgcaggggcaggcgctggtgcgggtgcctcgccctatagtgagtcgtattac |
|  | Reverse | 5’ gggccctttcgccattcaggctgcgca |
| Promoter for DsRed2 | Forward | 5’ gaattctaacaccgtgcgtgttgactattttacctctggcggtgataatggttgc  aagcacatatcgaaaggatagtcttgataacca |
|  | Reverse | 5’ gtcgacttaattaataaagttaatttttttttttatcgatacttatggttatcaagactatcct  ttcgatatgt |
| DsRed2-H | Forward | 5’ ttaatta**AAGGAGGACA**aatat**ATG**gcgagcagtgagaacgtcatcaccgagttcat |
| DsRed2-L | Forward | 5’ ttaatta**AAGGAGGCCA**gcctg**ATG**gcgagcagtgagaacgtcatcaccgagttcat |
|  | Reverse | 5’ ctcgagttacgccaccagggcatagttttcatcatttgccgccaggaacaggtggtggcg |

^a^ Restriction sites are underlined, and ATG start codon and RRS sequences are in uppercase letters.
